# Patient satisfaction after hook plate treatment of bony avulsion fracture of the distal phalanges

**DOI:** 10.1186/s40001-018-0332-y

**Published:** 2018-07-20

**Authors:** H. Vester, L. Schul, F. von Matthey, M. Beirer, M. van Griensven, S. Deiler

**Affiliations:** 1 Interdisciplinary Hand Department IHZ, Department of Trauma Surgery, Klinikum rechts der Isar, Technical University Munich, Munich, Germany; 2 Experimental Trauma Surgery, Department of Trauma Surgery, Klinikum rechts der Isar, Technical University of Munich, Munich, Germany

**Keywords:** Hook plate, Avulsion fracture, Self-assessment score, Mallet fracture

## Abstract

**Background:**

Bony avulsion fractures of the distal phalanges can result in mallet finger deformity if not treated appropriately. Therefore, only minimally displaced fractures can be treated conservatively with a good outcome, as dislocation occurs very often. Several surgical treatment options have been developed during the past decades. Data concerning the recently developed hook plate are promising. So far, no data concerning the subjective satisfaction with this method have been published. Therefore, we have analyzed the outcome after hook plate implantation using a self-assessment score, which focuses also on subjective parameters and satisfaction.

**Methods:**

Standardized questionnaires (self-assessment scores and SF-36 questionnaire) were sent to each patient treated with a hook plate due to fracture of the distal phalanx, type Doyle IVb and IVc. Clinical data were evaluated according to the medical record. Scores given per question range from 0 to 10, 10 is the worst and 0 the best outcome.

**Results:**

From 69 patients treated, 38 (58%) were enrolled. The whole collective (*n* = 38) reached a score of 39.7 ± 28.7 points, while men had slightly better results. Men (*n* = 24) achieved 37.3 ± 27.9 points, women (*n* = 14) 43.9 ± 30.7 points. Women had significantly better results when analyzed later than 12 months after surgery (52.1 ± 27.9 vs. 29.1 ± 32.8), whereas no changes could be detected in the male group (37.1 ± 29.9 vs. 37.4 ± 27.6). Overall, men were slightly more satisfied than women. Most satisfaction was found regarding pain and fine motor skills (0–0.46 points). Esthetic aspect and nail deformities (3.65 points average) led to the highest dissatisfaction. No differences in the SF 36 score could be detected.

**Conclusions:**

The hook plate is not only a convenient method but it also results in high patient satisfaction. Nail deformities are challenging; however, with increasing experience of the surgeon they decrease. SF 36 score is not an appropriate testing tool for this problem.

**Electronic supplementary material:**

The online version of this article (10.1186/s40001-018-0332-y) contains supplementary material, which is available to authorized users.

## Background

Traumatic mallet fractures are avulsion fractures of the distal phalangeal base including peri- or intraarticular fractures comprising the insertion of the extensor tendon. Indication for surgery depends on the fragment size and its dislocation, as well as a possible dislocation of the distal phalanx. These fractures are classified according to the Doyle classification. Fragments less than 50% of the articular joint (Doyle IVb) without dislocation could be treated conservatively with good results. However, in case of fragment dislocation (Doyle IVc) or dislocation of the distal phalanx surgical treatment is indicated [1–3].

**Fig. 1:**
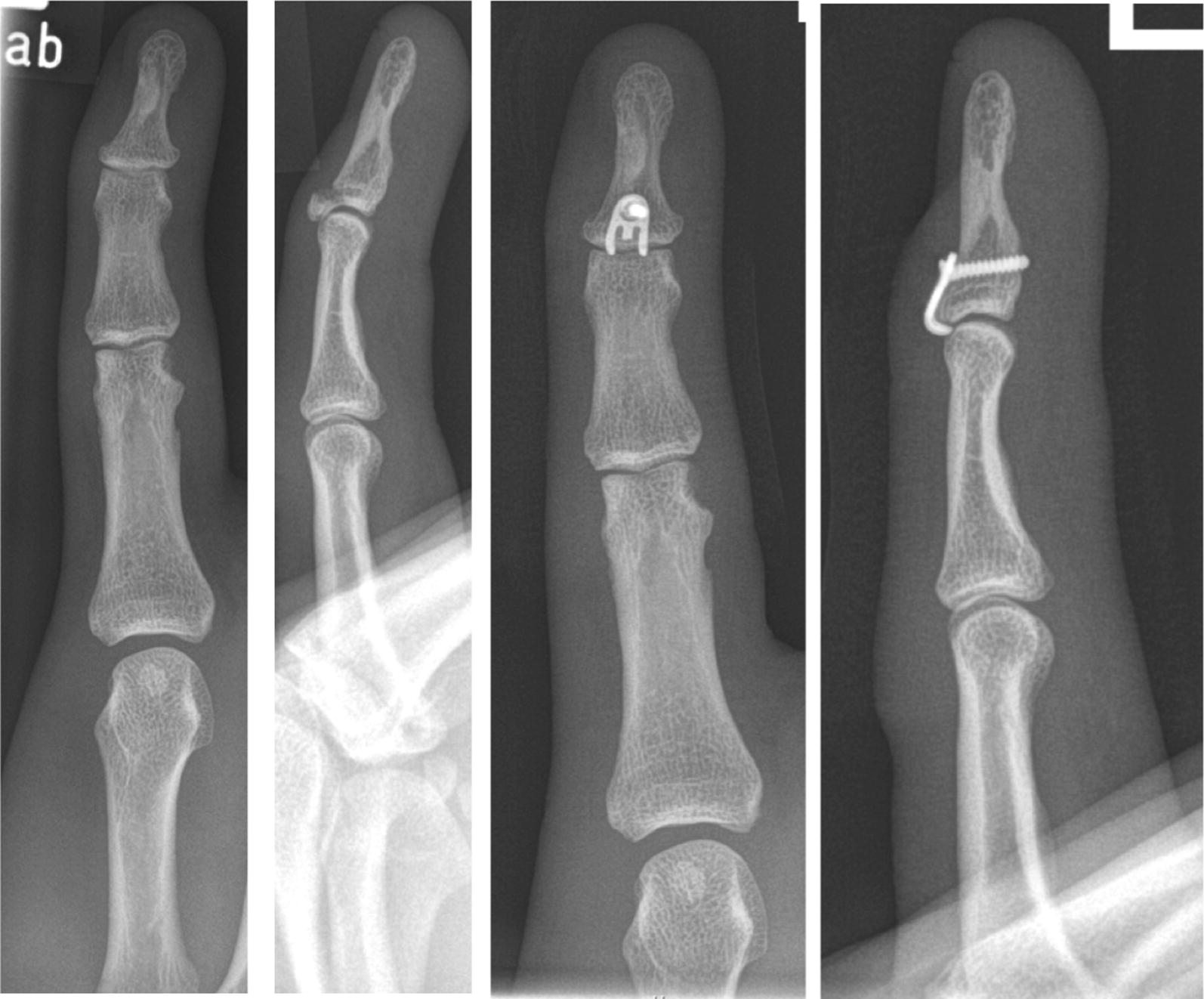
A distal phalanx fracture with dislocation and the postoperative x-ray after treatment with a hook plate

Several treatment options for the bony mallet finger have been developed during the past decades [14–16]. These are for example k-wire fixation [3], percutaneous extension block wiring [4, 5], screw osteosynthesis [6], compression pins [7], pull-through wires, figure of eight wiring [8, 9], tension band wiring [8, 10, 11], and umbrella handle k-wire fixation [12, 13]. However, these methods achieve only poor reduction and are associated with unsatisfactory outcomes and several complications [2, 17]. Temporary k-wire arthrodesis of the DIP, for example, increases the risk of DIP arthrosis and cartilage injury due to drilling heat and the damage done by the k-wiring. To avoid these problems, microscrew fixation has been tried [6, 18]. It allows a closed, rigid fixation without joint damage. However, small fragment are often fractured by the microscrew which makes a fixation impossible. Therefore, the hook plate has been developed [2, 17]. The first results of hook plate fixation for mallet fractures/Doyle IVc published seem promising [17, 19] (Fig. 1). There are a few studies published concerning functional outcome after hook plate fixation of avulsion fractures of phalanges. Aim of this study was to analyze the subjective and objective outcome and the patients’ satisfaction with this fixation method. In contrast to the preceding publications, this study has focused not only on objective parameters concerning the outcome but also on subjective patient satisfaction.

## Methods

From 2012 to 2015, 69 patients with a distal avulsion fracture were treated with hook plate fixation. The inclusion criteria included age > 18 years, fracture of the distal phalanx, fracture type Doyle IVb and IVc which were treated with hook plate fixation and willingness to give their informed consent to participation. Standardized questionnaires (self-assessment scores and SF-36 questionnaire) were sent to each patient postoperatively after having given their informed consent; clinical data, e.g., patient age, affected finger, and gender, were evaluated according to the medical record. The local ethical review committee of the Faculty of Medicine of the Technical University of Munich approved this study (369/14). The study was performed according to the Declaration of Helsinki in its newest version.

The self-assessment score was sent to the patients 3 months postoperatively at the earliest. It consists of 24 questions concerning individual satisfaction and range of movement (Table 1). Scores given per question range from 0 to 10, 10 is the worst and 0 the best outcome. Thus, the lower the maximum score the better the result (Additional file 1).

**Table 1:** The five best and worst rated items of the whole collective

Patients	Five best rated items	Five worst rated items
All patients	8, 6, 3, 7, 14	19, 11, 24, 17, 22
< 12 months postop	8, 7, 6, 3, 14	11, 19, 24, 17, 5
≥ 12 months postop	8, 6, 3, 14, 7	19, 17, 22, 11, 24

1. Intensity of pain, 4 weeks		
2. Most intense pain, 4 weeks		
3. Current intensity of pain		
4. Frequency of pain, 4 weeks		
5. Stress-induced pain		
6. Rest pain		
7. Pain-related sleeping problems		
8. Frequency of intake of painkillers		
9. Paraesthesia		
10. Swelling		
11. Nail deformity		
12. Typing on a keyboard		
13. To do up/undo one’s button		
14. To lace up one’s shoes		
15. Affection of work life		
16. Affection of doing one‘s hobbies		
17. Overall function		
18. Images (➔ extension deficit)		
19. Cosmetic result		
20. Worries		
21. Quality of life		
22. Overall treatment result		
23. Redecision for hook plate in case of another accident		
24. Annoyance by hook plate (if in situ)		

While pain intensity (during movement, during sleep, while rest) was low and painkiller intake was seldom, patients were unsatisfied with the cosmetic result and the stress-induced pain

Furthermore, the SF-36 life quality score was sent to all patients as well. Questionnaire evaluation was performed according to the SF-36 instructions.

## Results

### Epidemiology

**Fig. 2:**
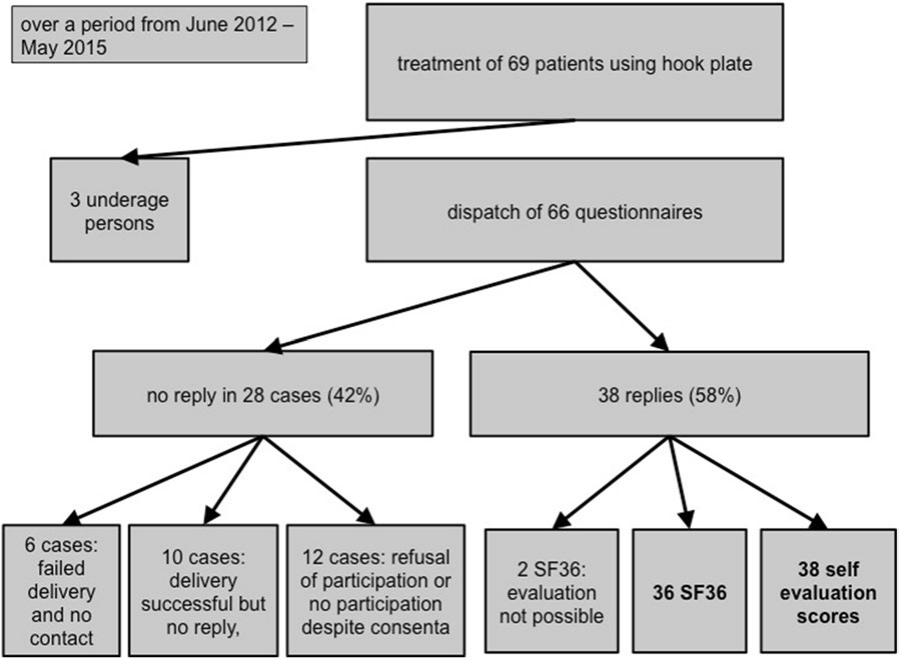
The patients’ enrolment and the return rate of the self-assessment scores

Overall, we treated 69 patients with a hook plate due to a distal phalanx avulsion fracture. Three of them were younger than 18 years and excluded from the study. Of the remaining 66 patients, 28 (42%) had to be excluded because 6 were not reachable, 10 did not answer the questionnaire and 12 refused participation. Thus, 38 patients (58%) were enrolled, 38 questionnaires, and 36 SF 36 scores could be evaluated (Fig. 2).

Overall, the gender ratio was male:female 24:14; the mean age was 38.1 years (range 20–77), in 12 patients (m:f = 7:5) the hook plate had already been removed. 19 patients were analyzed 3–12 months and 19 patients were analyzed more than 12 months after implantation of the hook plate (Table 2).

**Table 2:** This table depicts the patient collective before and after metal removal

	Male	Female	Male + female
Count	24 (63%)	14 (37%)	38 (100%)
Ø age	38.1 (20–77)	36.8 (23–74)	37.6 (20–77)
Removal of hook plate	8 (33%)	6 (43%)	14 (37%)

In 60.5% of the patients, the fifth finger was involved (*n* = 23), in 13.2% the third and ring finger (*n* = 5 each), in 10.5% the index finger (*n* = 4) and in 2.6% (*n* = 1) the thumb (Table 3).

**Table 3:** The distribution of the injured fingers

	1		2		3		4		5	
Count (L/R)	1	0	0	4	4	1	2	3	9	14
L + R	1 (2.6%)		4 (10.5%)		5 (13.2%)		5 (13.2%)		23 (60.5%)	
Mean score	26.3		39.3		34.6		33.3		42.39	
SF36 PHYSu/PSYSu	50.22/32.95		53.59/55.47		55.29/45.53		52.77/51.91		54.43/51.52	

The left side was in 16 (42%) and the right side in 22 (58%) patients injured. The little finger (60.5%) was affected most frequently

### Self-assessment score

The whole collective (*n* = 38) reached a score of 39.7 ± 28.7 points. Men (*n* = 24) achieved 37.3 ± 27.9 points, women (*n* = 14) 43.9 ± 30.7 points. Metal removal did not change the score in the male group (*n* = 7) 36.7 ± 17.7 before vs. 37.5 ± 31.7 points after metal removal and significantly in the female group (*n* = 5) 49.8 ± 23.3 after vs. 40.6 ± 34.0 before metal removal. The whole collective before metal removal had an overall score of 38.4 ± 31 points (*n* = 26) (Table 4).

**Table 4:** This table depicts the results of the self evaluation questionnaires

	Male Mean score (SD) (*n*)	Female Mean score (SD) (*n*)	Male + female Mean score (SD) (*n*)
Mean score	37.3 (27.9) (24)	43.9 (30.7) (14)	39.7 (28.7) (38)
After removal of HP	36.7 (17.7) (7)	49.8 (23.2) (5)	41.6 (20.6) (12)
Before removal of HP	37.5 (31.7) (17)	40.6 (34.0) (9)	38.4 (31) (26)
< 12 months postop	37.1 (29.9) (10)	52.1 (27.9) (9)	44.2 (29.2) (19)
≥ 12 months postop	37.4 (27.6) (14)	29.1 (32.8)(5)	35.2 (28.3) (19)

Moreover, there were no differences in score in the male group concerning the time after implantation of the hook plate (< 12 months vs. ≥ 12 months: 37.1 ± 29.9 vs. 37.4 ± 27.6). However, women had significant better scores 12 months after surgery compared with those analyzed earlier (29.1 ± 32.8 vs. 52.1 ± 27.9) (Table 4).

Overall, men (*n* = 24) were slightly more satisfied with the hook plate treatment than women (*n* = 14) (37.3 ± 27.9 vs. 43.9 ± 30.7) (Table 4).

Regarding the single questions, the most satisfaction was found regarding pain (0.05 points average), pain killer intake (0 points), paraesthesia (0.81 points), and fine motor skills such as buttoning the shirt (0.68 points) or tying the shoelaces (0.46 points). The highest score, which means the highest dissatisfaction, could be found regarding the esthetic aspect (4.32 points), nail deformities (4.16 points), and the bulky appearing of the finger due to the plate with an overall feeling of impairment (3.65 points average) (Table 1).

**Fig. 3:**
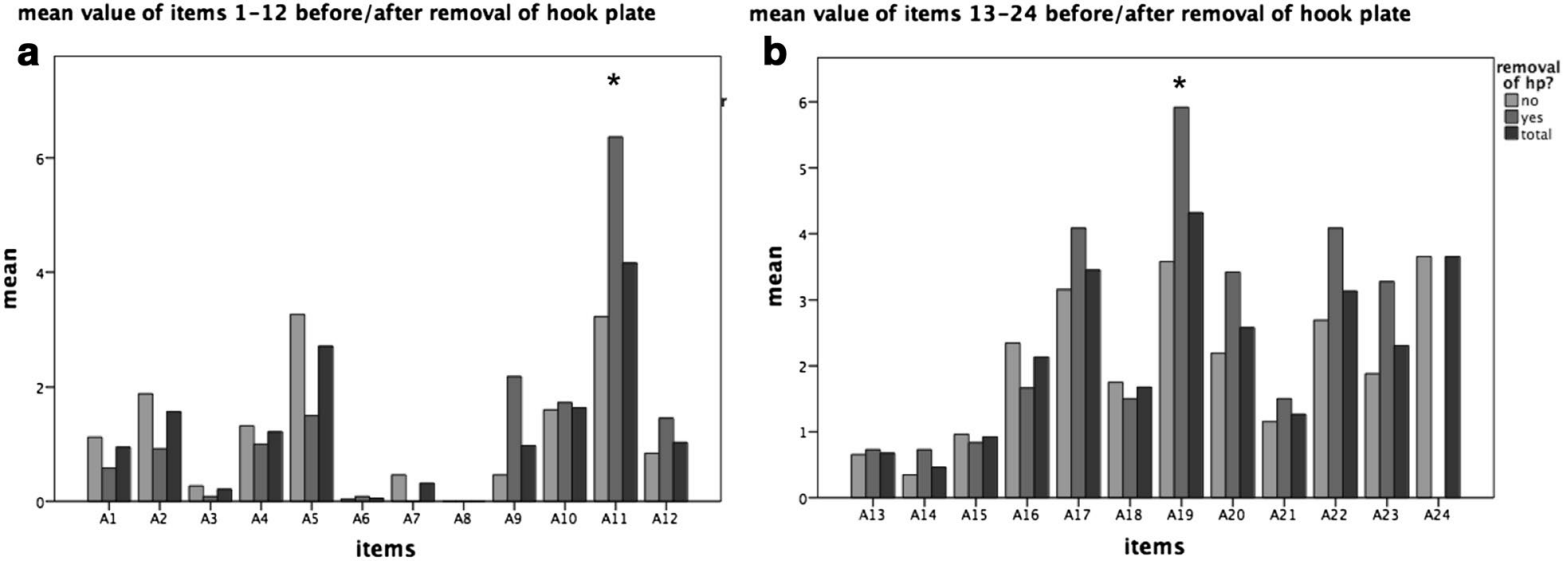
The mean scores before and after hook plate removal (**a** items 1–12, **b** items 13–24). No significant changes could be found in the whole collective, except a significant higher dissatisfaction regarding cosmetic result and nail deformities after metal removal compared to the patients with implanted hook plate. However, the stress-induced pain was reduced after hook plate removal. A11 nail deformity *p < 0.05, Man Whitney *U* test. A19 cosmetic result *p 0.05, Man Whitney *U* test

Regarding the outcome after metal removal, no significant changes could be found in the whole collective, except a significant higher dissatisfaction regarding cosmetic result and nail deformities after metal removal compared to the patients with implanted hook plate. However, the stress-induced pain was reduced after hook plate removal (Fig. 3).

**Fig. 4:**
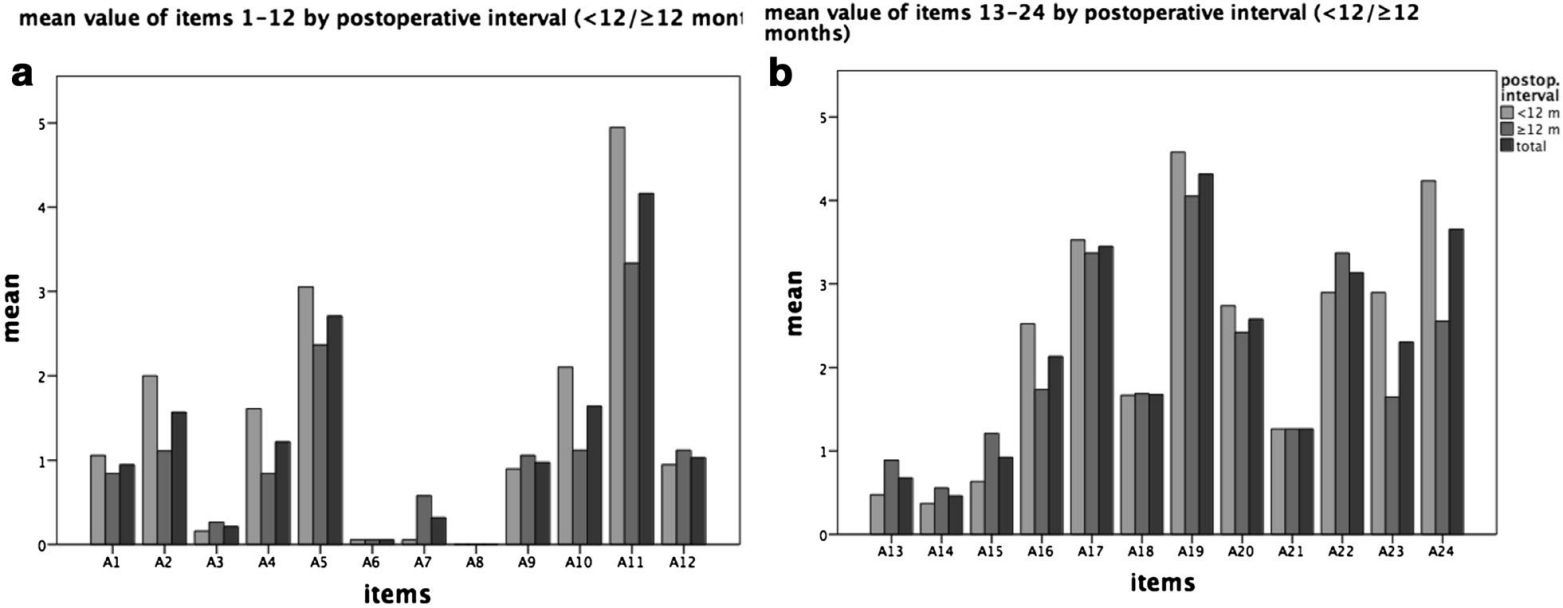
The mean scores related to the postoperative interval (**a** items 1–12, **b** items 13–24). No significant changes could be detected regarding the time except nail deformity and annoyance concerning the plate. The period of time after surgery has no significant impact on the subjective satisfaction

Moreover, no significant changes could be detected regarding the time except nail deformity and annoyance concerning the plate. The period of time after surgery has no significant impact on the subjective satisfaction (Fig. 4, Table 3).

### SF-36 life quality score

Regarding the SF-36 life quality score, we could detect no differences in the whole collective. The mean SF 36 Score was about 50 points in men, women, before removal of the plate, after removal of the plate, within 12 months post surgery as well as later than 12 months after surgery. As 50 points is defined as the baseline, according to the mean score of a predetermined population, no changes in SF-36 score could be detected, although some patients were unsatisfied with the esthetic aspect of the hook plate (Table 5).

**Table 5:** In this table the results of the SF 36 score are depicted

Scale	Male		Female		Male + female	
Mean score	53.40 (5.44) (23)	49.60 (7.50) (23)	55.38 (3.09) (13)	52.56 (6.18) (13)	54.11 (4.78) (36)	50.67 (7.11) (36)
After removal of hook plate	56.12 (4.16) (6)	45.26 (5.75) (6)	55.50 (3.53) (4)	51.84 (3.57) (4)	55.87 (3.73) (10)	47.89 (5.84) (10)
Hook plate in situ	52.43 (5.62) (17)	51.13 (7.58) (17)	55.32 (3.11) (9)	52.89 (7.22) (9)	53.43 (5.02) (26)	51.74 (7.37) (26)
< 12 months	51.76 (5.45) (10)	50.54 (8.71) (10)	54.87 (3.06) (8)	52.71 (7.10) (8)	53.14 (4.70) (18)	51.51 (7.89) (18)
≥ 12 months	54.66 (5.30) (13)	48.87 (6.70) (13)	56.18 (3.31) (5)	52.33 (5.12) (5)	55.08 (4.79) (18)	49.83 (6.35) (18)

The mean score is shown with the standard deviation (SD) and number of patients in braces. In the first box the score of the physical health summary scale is depicted and in the second box the score of the mental health summary scale is shown. No significant differences could be detected

## Discussion

The avulsion of the terminal extensor tendon from the base of the distal phalanx with or without a bony fragment is called a mallet finger. The disruption of the terminal extensor tendon results in the characteristic flexion deformity of the distal interphalangeal joint. Axial loading or a forceful flexion of the extended digit, as it can be found typically in ball-related sports injuries, is the common trauma mechanisms [20]. Inadequate treatment results in extensor lag, early osteoarthritic changes of the distal interphalangeal joint, or even a swan neck deformity [1, 21].

Treatment options include conservative strategies and surgical procedures. Some authors suggest surgery for avulsion fractures when more than one-third of the articular surface is involved as well as for subluxation or fragment displacement which cannot be reduced in a finger splint completely extending the distal interphalangeal joint [22]. Other authors advocate conservative treatment with splint fixation. However, due to the high complication rate during conservative treatment like losing reduction, and reduced flexion of the distal interphalangeal joint [1], the authors advocate surgical treatment of all bony lesions already with only minimal displacement.

Aims of the surgical treatment are restoration of a congruent articular arc without subluxation and of an anatomical reduction and fixation of the bony fragment to prevent joint deformity, post-traumatic arthrosis and stiffness. Several different surgical techniques exist and have been discussed in the literature: open reduction and k-wire fixation [23], tension band wire [10, 11], tenodermodesis [24], pull-out steel wires [11] and screw fixation [6]. Percutaneous pin fixation [16, 25], percutaneous extension block pinning [26] and percutaneous compression fixation pins [18] and the so-called fish hook technique [27] have also been tried to minimize surgical complications while improving fragment reduction.

Although numerous different surgical techniques exist, the postoperative complication rate in general has been rather high, ranging from 3 to 55% depending on the study cited [19, 28, 29]. However, the recently developed hook plate showed promising results. Unfortunately, the few studies published deal only with a small number of patients, i.e., *n* = 9 [2] or *n* = 13 [30]. Szalay et al. [17] were the first with a higher number of 59 patients. In their study, nail deformity occurred in 12% using the Stryker hook plate. Teoh et al. also used the Medartis^®^ hook plate and reported nail deformities in 23% (3 patients out of 9) [2]. We had a collective of 38 patients enrolled and nail deformity was also the most criticized aspect of this treatment. In fact, it was one of the subjectively most criticized aspects of this treatment followed immediately by the unhappiness concerning the esthetic aspect of the distal phalanx due to the bulking of the plate. However, we noticed a certain training curve concerning the nail deformities, as their frequency became less over time. Like Teoh and Lee [2], we recommend removal of the plate 3–6 months after implantation. However, not all patients wanted a plate removal because they did not feel annoyed by it.

So far, the outcome of the treatment with a hook plate has not been analyzed concerning the subjective satisfaction of the patients. Studies could show good to excellent radiological and clinical outcomes with complete bony consolidation and proper plate positioning as well as good tendon function and proper movement of the finger. This study has focused on the subjective outcome of the patient concerning the individual satisfaction with the surgery.

Moreover, the time period after implantation has no decisive impact on the subjective satisfaction except, once again nail deformity and subjective annoyance by the hook plate. Both issues were rated worse from the patients within the first 12 months after surgery. One reason for that might be that the patients asked to a later time were already used to the plate and temporary nail deformities might have already healed.

After removal of the hook plate, the self-assessment score concerning stress-induced pain got significantly better while subjective satisfaction concerning cosmetic outcome and nail deformity got significantly worse. This might be due to the high expectations patients have concerning the metal removal. Often, nail deformities are due to an injury of the nail bed, which might be irreparably injured or healing takes a long time and cannot be accelerated by metal removal. Moreover, postoperative swelling and reddening of the distal phalanx might still have been present. Finally, some patients still have a deficiency concerning maximum extension of the finger.

The SF-36 score is widely acknowledged as a life quality score used generally in all studies concerning satisfaction and personnel happiness after trauma or surgery of the upper extremities [31, 32]. However, it does not seem to be a reasonable questionnaire for analyzing this special outcome in the phalangeal area. It is far too unspecific as only one finger is concerned. In our collective, we could find more or less no difference in the scores of our collective compared with the standardized comparison group of the American individual. All individuals achieved around about 50 points. Regarding the single questions in detail, not all of them were really satisfied with the finger, which of course also has a certain impact on life quality.

However, this study has some limitations. First, we could enroll only 44 patients, which is good compared to other published studies, but further studies with more patients are needed for improvement of statistical power. Moreover, we could show only a few significant results, which might be due to the small patient number.

## Conclusions

In conclusion, the hook plate is a reliable and safe method for treatment of bony avulsion fractures of the distal phalanx as several authors have shown before. Concerning the subjective satisfaction of the patient, the results [2, 17, 30] are good to excellent with only two deficits, which are the cosmetic outcome and the nail deformity. Patients should be informed about this during the informative conversation before giving their informed consent.

## Additional file

**Additional file 1.** Finger self-assessment questionnaire.
