# Supplementary material for: Patient satisfaction after hook plate treatment of bony avulsion fracture of the distal phalanges
Source: Eur J Med Res. 2018 Jul 20;23:35. doi: 10.1186/s40001-018-0332-y (PMC6053819; doi:10.1186/s40001-018-0332-y)
Supplement: Supplementary file 1 — Additional file 1. Finger self-assessment questionnaire. [file 40001_2018_332_MOESM1_ESM.pdf]

[redacted]

Interdisziplinäres Handzentrum (IHZ)
Leitung: Dr. Stephan Deiler
Klinik und Poliklinik für Unfallchirurgie
Direktor: Univ.-Prof. Dr. Peter Biberthaler

[redacted]

Patienten ID

Datum:

Geschlecht: ☐ weiblich ☐ männlich

Geburtsdatum:

Welcher Finger ist betroffen?

☐ rechter
☐ linker

☐ Daumen
☐ Zeigefinger
☐ Mittelfinger
☐ Ringfinger
☐ kleiner Finger

Sind Sie berufstätig?

☐ ja ➔ Ihr Beruf:______________________________
☐ nein ➔ aufgrund Ihrer Fingerverletzung? ☐ ja ☐nein
☐ Rentner
☐ Hausfrau

Nehmen Sie Medikamente zur Linderung der Beschwerden an Ihrem Finger ein?

☐ ja
☐ nein

falls „ja“, welche Medikamente?

Womit beschäftigen Sie sich in Ihrer Freizeit? Was sind Ihre Hobbys?

[redacted]

Interdisziplinäres Handzentrum (IHZ)
Leitung: Dr. Stephan Deiler
Klinik und Poliklinik für Unfallchirurgie
Direktor: Univ.-Prof. Dr. Peter Biberthaler

[redacted]

1. Wie stark waren die Schmerzen an Ihrem Finger den vergangenen 4 Wochen?

0	1	2	3	4	5	6	7	8	9	10

keine Schmerzen — unerträglich

2. Wie stark waren die größten Schmerzen an Ihrem Finger in den vergangenen 4 Wochen?

0	1	2	3	4	5	6	7	8	9	10

keine Schmerzen — unerträglich

3. Wie stark sind die Schmerzen an Ihrem Finger in diesem Moment?

0	1	2	3	4	5	6	7	8	9	10

keine Schmerzen — unerträglich

4. Wie häufig hatten Sie in den vergangenen 4 Wochen Schmerzen an Ihrem Finger?

0	1	2	3	4	5	6	7	8	9	10

nie — immer

5. Haben Sie unter Belastung Schmerzen an Ihrem Finger?

0	1	2	3	4	5	6	7	8	9	10

nie — immer

6. Haben Sie in Ruhe Schmerzen an Ihrem Finger?

0	1	2	3	4	5	6	7	8	9	10

nie — immer

7. Konnten Sie aufgrund von Schmerzen an Ihrem Finger in den vergangenen 4 Wochen schlechter schlafen?

0	1	2	3	4	5	6	7	8	9	10

nie — immer

8. Wie oft nehmen Sie Schmerzmittel gegen Ihre Schmerzen am Finger ein?

0	1	2	3	4	5	6	7	8	9	10

nie — immer

9. Leiden Sie unter Gefühlsstörungen an Ihrem Finger wie Taubheit/Kribbeln/Einschlafen/Pelzigsein?

0	1	2	3	4	5	6	7	8	9	10

nie — immer

10. Ist Ihr Finger im Vergleich zum Zustand vor der Behandlung geschwollen?

0	1	2	3	4	5	6	7	8	9	10

keine Schwellung — extreme Schwellung

11. Bemerken Sie Veränderungen am Fingernagel des betroffenen Fingers im Vergleich zum Zustand vor der Behandlung?

0	1	2	3	4	5	6	7	8	9	10

überhaupt nicht — ja, sehr

12. Mein Finger behindert mich beim Tippen auf einer Tastatur.

0	1	2	3	4	5	6	7	8	9	10

nie — Tippen unmöglich

13. Mein Finger behindert mich beim Öffnen oder Schließen von Knöpfen an meiner Kleidung.

0	1	2	3	4	5	6	7	8	9	10

nie — Öffnen/Schließen unmöglich

14. Mein Finger behindert mich beim Schuheschnüren.

0	1	2	3	4	5	6	7	8	9	10

nie — Schnüren unmöglich

[redacted]

Interdisziplinäres Handzentrum (IHZ)
Leitung: Dr. Stephan Deiler
Klinik und Poliklinik für Unfallchirurgie
Direktor: Univ.-Prof. Dr. Peter Biberthaler

[redacted]

15. Behindert Sie ihr Finger bei der Arbeit?

0	1	2	3	4	5	6	7	8	9	10

nie immer

16. Behindert Sie Ihr Finger beim Ausüben Ihrer Hobbys (Musik/Sport etc.)?

0	1	2	3	4	5	6	7	8	9	10

nie immer

17. Wie zufrieden bzw. unzufrieden sind Sie mit der allgemeinen Funktionsfähigkeit Ihres Fingers?

0	1	2	3	4	5	6	7	8	9	10

sehr zufrieden äußerst unzufrieden

18. Bitte kreuzen Sie jede Ihnen ohne Hilfsmittel einnehmbare Position an.

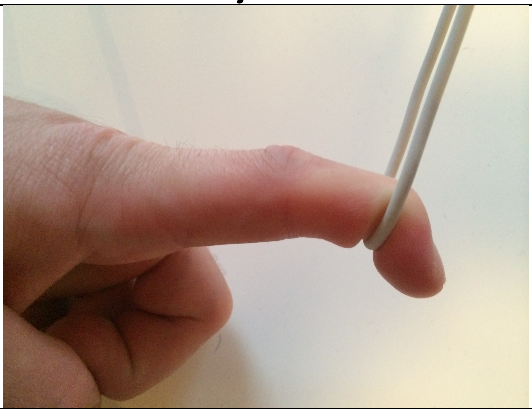	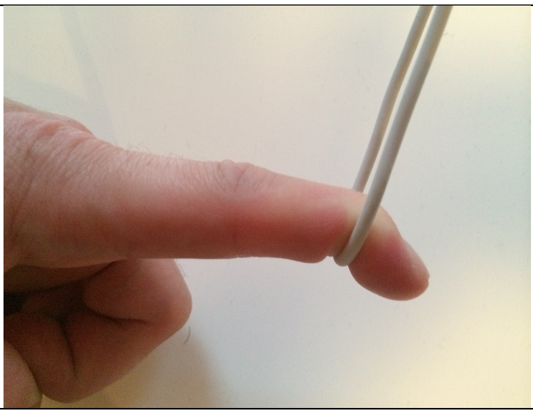
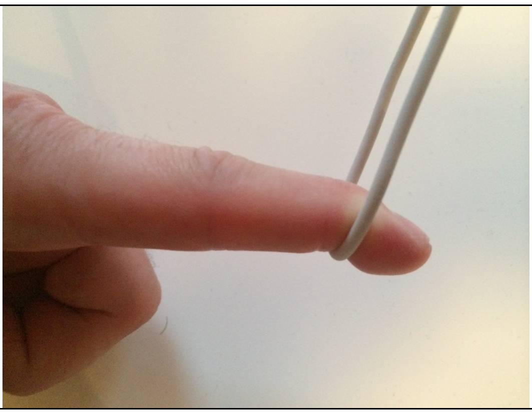	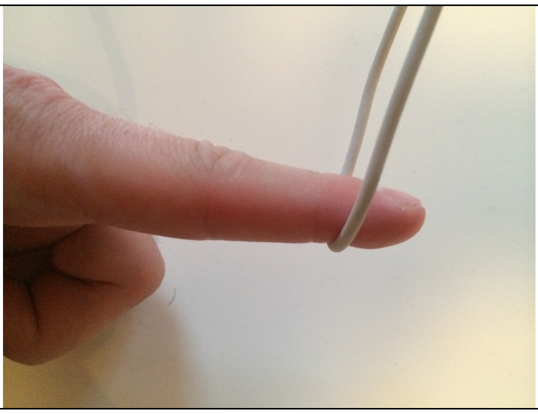

19. Wie zufrieden bzw. unzufrieden sind Sie mit dem Aussehen Ihres Fingers nach der Behandlung?

0	1	2	3	4	5	6	7	8	9	10

sehr zufrieden äußerst unzufrieden

20. Machen Sie sich bezüglich Ihres Fingers Sorgen?

0	1	2	3	4	5	6	7	8	9	10

nie immer

21. Sind Sie wegen Ihres Fingers in Ihrer Lebensqualität eingeschränkt?

0	1	2	3	4	5	6	7	8	9	10

überhaupt nicht ja, sehr

22. Wie zufrieden bzw. unzufrieden sind Sie mit dem Ergebnis der Behandlung?

0	1	2	3	4	5	6	7	8	9	10

sehr zufrieden äußerst unzufrieden

23. Würden Sie sich im Falle einer erneuten Verletzung wieder für die bei Ihnen vorgenommene Behandlungsmethode entscheiden?

0	1	2	3	4	5	6	7	8	9	10

immer niemals

24. (Falls die bei Ihnen eingesetzte Hakenplatte noch nicht entfernt wurde:) Fühlen Sie sich durch das an Ihrem Finger angebrachte Fremdmaterial beeinträchtigt?

0	1	2	3	4	5	6	7	8	9	10

nie immer
